# Exploring the relationship between blood platelet and other components utilizing count regression: A cross‐sectional study in Bangladesh

**DOI:** 10.1002/hsr2.70007

**Published:** 2024-08-20

**Authors:** Umme Honey, SM Arif Bin Saleh, Md. Sifat Ar Salan, Mohammad Alamgir Kabir, Akher Ali

**Affiliations:** ^1^ Department of Statistics and Data Science Jahangirnagar University Dhaka Savar Bangladesh; ^2^ NYU Langone Health Long Island New York USA

**Keywords:** blood platelet, negative binomial regression, quasi‐Poisson regression, white blood cell (WBC)

## Abstract

**Background and Aims:**

Blood, vital for transporting nutrients and maintaining balance, comprises red blood cells, white blood cells, and platelets, each pivotal. Imbalances lead to issues—low red cells cause fatigue (anemia), high white cells hint at infection, low counts raise infection risks. Using trendy statistical approaches, investigating the complex link between platelet counts and numerous blood components. Our investigation, leveraging count regression approaches, revealed deep insights into the interaction between platelet counts and other important hematological markers.

**Methods:**

A cross‐sectional study utilized data from 3120 individuals, including both male and female participants, who visited these hospitals between June 16, 2022 and December 17, 2022, to assess their blood samples through testing by using convenience non‐parametric sampling framework. Platelet count was taken into account as a measure of outcome in this research. This specific study region was chosen for its easy accessibility, which helped the seamless execution of the data‐gathering technique. Count regression, negative binomial regression, and quasi‐Poisson regression techniques have been employed for examining relationship of the data sets.

**Results:**

Three different count regression models were utilized to assess the proper association between the response and the relevant covariates and we found negative binomial count regression model (Akaike information criterion = 76.55, Bayesian information criterion = 76.59, and deviance = 3.14) was providing comparatively better performance than others. Based on the chosen model we found white blood cell, erythrocyte sedimentation rate, and eosinophils are significant but neutrophil, monocyte, and lymphocyte are not significant. We have also gone through proper model adequacy checking for our selected model and we found enough evidence to justify our model.

**Conclusion:**

From the result, we found insightful remarks into the mechanisms involved in platelet production and regulation, which can aid in developing increased effective treatments and interventions to maintain optimal platelet levels and prevent health problems related to abnormal platelet counts.

## 1 INTRODUCTION

Blood is an essential bodily fluid that plays critical roles in transporting nutrients, oxygen, and waste products, and maintaining homeostasis. It consists of primary components: red blood cells, white blood cells (WBCs), and platelets, each with unique and vital functions.^1^, ^2^ Imbalances in these components can lead to a variety of health issues. For instance, a low red blood cell count can result in anemia, characterized by fatigue and shortness of breath. Similarly, WBC counts can indicate immune health, where high counts may suggest an infection and low counts increase infection risk. Platelet abnormalities can lead to clotting or bleeding disorders, such as thrombosis and thrombocytopenia.^3^

The connection between blood components and various medical conditions is well‐documented. High cholesterol and triglyceride levels are linked to cardiovascular diseases, while high blood sugar levels are associated with diabetes.^4^ Certain cancers can also impact blood cell production or cause abnormal blood cell counts.^5^, ^6^ The significance of maintaining optimal platelet levels is highlighted by studies showing that thrombocytopenia (low platelet count) can cause bleeding and clotting disorders, whereas thrombocytosis (high platelet count) can increase the risk of cardiovascular diseases and thrombosis.^7^ Platelets interact with red and WBCs and endothelial cells to manage blood thickness, immune responses, and blood vessel dilation.^8^ WBCs, complete blood count (CBC), erythrocyte sedimentation rate (ESR), thrombocytes, eosinophils, and basophils are among the other blood components that affect their levels. For example, WBCs are positively linked to platelet counts in rheumatoid arthritis patients, and CBC is associated through platelet counts in individuals suffering from chronic obstructive pulmonary disease and liver cirrhosis.^9^, ^10^

Despite the extensive research on individual blood components and their associated health issues, there is a lack of comprehensive studies examining the complex interactions between these components, particularly how they collectively influence platelet levels. Most existing studies focus on singular associations rather than an integrative approach that considers multiple blood indicators simultaneously. This gap in research limits our understanding of the underlying mechanisms that regulate platelet production and maintenance. Furthermore, the predictive models that explore these interactions are underutilized, leaving significant potential for advancements in early detection and preventive healthcare unaddressed

This study aims to fill the research gap by using the count regression model to understand the relationships between platelet levels and various blood components, including WBCs, CBC, ESR, thrombocytes, eosinophils, and basophils. By identifying significant predictors and their influence on platelet counts, we seek to provide information regarding the mechanisms governing platelet production and regulation. This understanding will assist in the invention of more effective treatments and interventions to maintain appropriate platelet levels of difficulty, therefore preventing health complications associated with aberrant platelet counts. Ultimately, our research aims to enhance early detection and preventive measures for conditions linked to platelet abnormalities, improving overall health outcomes.

## 2 METHODS

### 2.1 Research design and context

The context of this research is to signify the other blood components that are causing serious changes in the platelets count of the patients with different blood‐related diseases of the geographical area of Savar, Dhaka.

The study uses a quantitative research design with a focus on count regression models to analyse the relationship between platelet count and various blood parameters. The design is cross‐sectional, analyzing a sample of 3120 observations collected at a single point in time from four local hospitals. This design allows for the examination of associations between variables but does not assess causality.

### 2.2 Data and variables

The study used quantitative methods to investigate how platelet count relates to other blood components such as WBCs, ESR, eosinophils, neutrophils, monocytes, and lymphocytes. In our country, medical reports do not have a centralized source, which highlights the importance of conducting related studies due to the prevalence of platelet‐related diseases. Nonetheless, data collection in vast areas can be challenging. Participants who visited hospitals for blood tests presented with symptoms including chest pain, heart palpitations, difficulty breathing, dizziness, vision changes, weakness, numbness, slurred speech, transient ischemic attacks, extreme fatigue, headaches, leg pain, and swelling, as well as an enlarged spleen or liver. Therefore, the researchers targeted Savar Upazila as their study area since it was a convenient location to carry out their data collection procedure.

### 2.3 Ethical consideration

The study obtained logistical clearance from the esteemed Department of Statistics at Jahangirnagar University, Savar, Dhaka‐1342, under reference code: JU/STAT/2022/01. Participation in the research was contingent upon the willing consent of individuals (patients), signified by their acknowledgment through a formal permission form, ensuring ethical and informed analysis.

The ethical approval from the hospital authorities also taken and we acknowledged that a formal ethical approval letter has been issued by them that the data can be used only for the research purposes.

The study adopts a positivist approach, reflecting a belief in an objective reality that can be measured and analyzed through statistical methods. This perspective is appropriate given the use of quantitative regression models to explore relationships between platelet count and other blood parameters.

### 2.4 Determination of sample size and sampling techniques

Nonparametric convenience sampling has been applied to collect the data. Actually, we have collected the hematology reports of the patients of the diagnostic center of the hospitals in between June 16, 2022 and December 17, 2022 with the consent of the patients as well as with the ethical approval of the hospitals. Finally, we got 3120 data from the patients accumulated in all the hospitals. The data from several reputable hospitals in the region, including Rose Clinic and Diagnostic Center (1329), Lab Zone Specialized Hospital (821), Ibn Sina Hospital (651), and Popular Diagnostic Center (319), to obtain a sample of 3120 individuals, including both male and female participants, by using convenience sampling framework. Savar Upazila inhabitants usually visit these hospitals to test their blood samples, which is the reason for considering this sample representative for the Savar area.

### 2.5 Unit of analysis

The unit of analysis in this study is the individual blood sample. Each observation represents a unique instance of blood parameters measured, allowing for an in‐depth examination of the relationships between these parameters.

### 2.6 Validity and reliability

The study controlled for potential confounding variables such as age, gender, and other relevant health indicators. Outliers were identified through exploratory data analysis and addressed to minimize their impact on the results. The findings are generally applicable to similar clinical or health settings with comparable populations. However, the results should be interpreted with caution when applied to different contexts or patient populations.

Data collection procedures were standardized and consistent across all observations. The value of Cronbach's *α* = 0.80 which ensures the reliability of the data sets. The instruments and methods used for measuring blood parameters have been validated in previous studies, ensuring that the results are accurate and replicable.

### 2.7 Statistical tools and techniques

The data was entered into an Excel spreadsheet, with platelet count serving as the dependent variable. The analysis included nine independent variables: gender, age, ESR, neutrophil count, monocyte count, lymphocyte count, and eosinophil count. The data put in R programming language and processed as well as coded. The value of Cronbach's *α* = 0.80 which ensures the reliability of the data sets. Then descriptive statistics was performed here. The data was analyzed using selected count regression models, including Poisson regression, negative binomial regression, and quasi‐Poisson regression, to determine the best‐fit model for the data. The alternative hypothesis where components are significantly affecting on platelets counts which stated that two tailed of the hypotheses. The entire programs used to generate the results in this report are written in R studio, R version 3.5.1 and Microsoft excel by using “MASS,” “pscl,” “glm2,” “Ime4,” “speedglm,” “glmmTMB,” “nbinom,” “countneg,” and “VGAM” packages.

### 2.8 Poisson regression model

Count regression procedures, such as Poisson regression, can be used for evaluating the relationship between platelet count and other parts of the blood, compensating into consideration the non‐negative and discrete characteristics of the count information.^11^ By including various independent variables in the regression model, such as age, gender, and several varieties of WBCs, we can identify significant predictors of platelet count and quantify their impact.

The findings of such studies can have implications for clinical practice, such as improving the accuracy of determining and evaluating medical conditions related to platelet count.^12^ Additionally, understanding the relationship between platelet count and other blood components can also provide insights into the underlying mechanisms of these conditions and suggest potential avenues for future research and development of new treatments.^13^ The Poisson coefficient equation is an analytical tool for analyzing count numbers, such as the amounts of platelets in plasma or the counts of different parts of the blood. It presupposes that the variable that depends, *y*, has a Poisson distribution as well as is linearly connected to the separate variables, *x*:

$$\mathrm{lo}g\left(y\right)=\beta_{1}+\beta_{1}x_{1}+{\beta_{2}x}_{2}+\ldots +\beta_{p}x_{p}+\varepsilon ,$$

where $\beta_{0}$ is the intercept, $\beta_{1}$ to $\beta_{p}$ are the rates of the exposure variables $x_{1}$ to $x_{p}$, respectively, and log is the natural logarithm function.^14^

The Poisson regression equation implies us to determine the impact of each independent variable on the count of the dependent variable while accounting for other variables in the model. For instance, we could utilize Poisson regression to determine the connection between platelet count and other blood components while controlling for gender, age, and additional factors.

### 2.9 Negative binomial regression model

Negative binomial regression is another type of count regression applied to analyse the relationship between platelet count and other blood components. As a generalized linear model, it accounts for overdispersion, meaning the variance of the outcome variable indicating platelet count can exceed the average, a common feature in count data.^15^ The negative binomial regression model is comparable to the conventional Poisson regression model but incorporates an additional parameter, called the dispersion parameter, which accounts for the overdispersion. The equation for negative binomial regression is:

$$\log\left(E\left(Y\right)\right)=\beta_{0}+\beta_{1}X_{1}+\beta_{2}X_{2}+\ldots +\beta_{p}X_{p}+\varepsilon ,$$

where E(Y) represents the expected value of the outcome variable (platelet count),

$X_{1}$ to $X_{p}$ denote the exposure variables (such as gender, age, ESR, neutrophil count, monocyte count, lymphocyte count, eosinophil count, and basophil count), and $\beta_{0}$ to $\beta_{p}$ are the similar rates of regression. The negative binomial regression model estimates the exponentiated regression coefficients, known as incidence rate ratios (IRRs), which indicate the multiplicative effect of each exposure variable on the outcome variable. An IRR of one reflects no difference; an IRR exceeding one demonstrates an additive effect; and an IRR beneath one reflects a negative influence on the amount of platelets.^16^

Overall, using negative binomial regression can provide valuable perspectives into the connection between platelet count and other components of blood, taking into account the potential overdispersion in the data.

### 2.10 Quasi‐Poisson regression model

The quasi‐Poisson regression model is a statistical method employed to analyse count data that have overdispersion, meaning that their variance exceeds the mean. In this model, the expected count is represented as a linear blend of the predicting variables, and its logarithm is taken.^17^ The quasi‐Poisson equation involves the intercept, regression coefficients, and predictor variables, and describes the connection between the expected count and predictor variables. The variance of the count is proportional to the expected count, with the proportionality constant being the dispersion parameter.^18^ Both the regression coefficients and dispersion parameter are estimated using maximum likelihood estimation.

$$\ln\left(E\left(Y\right)\right)=\beta_{0}+\beta_{1}X_{1}+\beta_{2}X_{2}+\ldots +\beta_{p}X_{p}+\varepsilon ,$$

Var(Y)=θE(Y),

where ln(E(Y)) represents the natural logarithm function, E(Y) is the expected value of the count variable Y, $\beta_{0}$ is the intercept, $\beta_{1}\;$to $\beta_{p}$ are the regression coefficients for the predictor variables $X_{1}$ to $X_{p}$, Var(Y) is the variance of the count variable Y, θ is the dispersion parameter.

## 3 RESULTS

### 3.1 Exploratory data analysis

Table 1 provides an overview of descriptive statistics for multiple important variables, such as age, WBC count, neutrophil count, lymphocyte count, eosinophil count, platelet count, and ESR, based on a sample size of 3120 observations. For each variable, the highest and lowest values, along with quartiles and median values, are reported. These statistics serve as a useful tool to understand the central tendency, variability, and range of each variable, and to identify any potential outliers or extreme values in the data set.

**Table 1:** Summary statistics.

Variable	Observation	Min	First quartile	Median	Mean	Third quartile	Max.
Platelet	3120	10000	22,000	25,000	254,479	280,000	2,300,000
Age	3120	0.14	22	28	32.27	40	100
WBC	3120	1000	7600	8600	8900	9800	20,000
ESR	3120	4	20	31	36.97	48	132
Neutrophil	3120	3500	5800	6300	6266	6600	8000
Lym	3120	1100	2800	3200	3194	3700	34,000
Esi	3120	100	300	300	318.6	400	600
Mono	3120	100	200	200	225.7	200	500

Abbreviations: Esi, eosinophil; ESR, erythrocyte sedimentation rate; Mono, monocytes; WBC, white blood cell.

A data set including numerous factors pertaining to age, gender, and blood parameters like WBC, neutrophils, lymphocytes, monocytes, eosinophils, and platelet count appears to be summarized in Table 1. Participants in the data include both male and female, with a mean age of 32.27 years. The summary demonstrates the substantial variability of the ESR, WBC, and platelet count in the sample, with a wide range of values recorded. Neutrophil and lymphocyte counts are within the normal range, while monocyte and eosinophils count have high variability. This overview as a whole indicates that the data set may be helpful in researching the connections between these blood markers and different health consequences.

**Figure 1:**
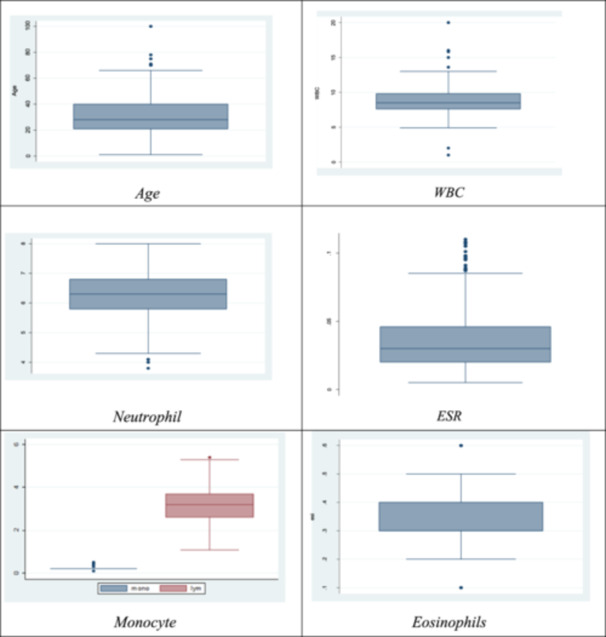
Boxplot of the selected distribution. ESR, erythrocyte sedimentation rate; WBC, white blood cell.

We have several options for describing data with univariate data. By drawing boxplot, we are trying to describe the data. A box plot, sometimes known as a boxplot, is a technique used in descriptive statistics to visually represent numerical data sets based on their quartiles. Box and Whisker plots and box and Whisker diagrams are named after the possibility of lines emerging from the boxes in the plot that show variability outside the upper and lower quartiles (Figure 1).

The data set provided contains various univariate distributions which are represented through box plots. Each box plot showcases a particular variable like age, WBC, ESR, neutrophil, platelet, monocyte, and eosinophils, and displays significant highlights of the information, such as the median, highest and lowest values, and the existence or not existence of outliers.

For instance, the data is symmetrical, with most observations cantered around the median, and the age box plot shows only one outlier. Likewise, there are no outliers displayed in the platelet box plot, and the data is symmetrical. Conversely, the ESR box plot indicates three outliers, and the data is skewed towards the right, with the majority of observations were cantered at the lower end of the range.

### 3.2 Model fitting

Table 2 presents a comparison of three commonly used count regression models: Negative binomial, Poisson, and quasi‐Poisson, which are often utilized to investigate count data representing the number of events that occur within a specific time period. Each independent variable in the models has its estimated coefficients listed in the table, along with the matching p‐values that signify the degree of statistical significance. A low *p*‐value indicates a significant effect of the exposure variable on the outcome variable. The expected coefficients and *p*‐values for each model and exposure variable are supplied separately, allowing you to assess how well they match the data.

**Table 2:** Summary of findings of different count regression models (Poisson regression, negative binomial regression, and generalized negative binomial regression model).

	Poisson regression model		Negative binomial regression model		Quasi‐Poisson regression model	
	Estimate	*p*‐Value	Estimate	*p*‐Value	Estimate	*p*‐Value
(Intercept)	12.26	0.0002	12.26	0.0002	12.26	*p* < 0.001***
WBC	0.0115	0.0002	0.0119	0.0201	0.0115	*p* < 0.001***
Neutrophil	0.0509	0.0667	−0.0309	0.9996	0.0590	*p* > 0.95
Monocyte	0.0405	0.5380	0.0804	0.9905	0.0405	*p* > 0.99
Lymphocyte	0.0624	0.0002	0.0689	0.9223	0.0624	*p* > 0.93
ESR	0.0225	0.0002	0.0222	0.0201	0.0225	*p* > 0.24
Eosinophils	0.0222	0.0002	0.0022	0.0003	0.0222	*p* < 0.001***

Abbreviations: ESR, erythrocyte sedimentation rate; WBC, white blood cell.

### 3.3 Model comparison

Akaike information criterion (AIC) and Bayesian information criterion (BIC) are useful tools for assessing the correlation between platelet count and other components of blood using count regression. Specifically, when comparing Poisson and AIC and BIC are useful tools for assessing the connection between platelet count and other components of blood using count regression. Specifically, when comparing Poisson and negative binomial regression models, AIC and BIC can help determine which model is the best fit for the data.^19^ The comparison process involves starting with a model without any exposure variables and gradually increasing independent exposures until reaching the entire model. The model with the minimum AIC is considered the best fit for the data.^20^ Using these penalized‐likelihood criteria can provide valuable insights into the relationship between platelet count and other components of blood, allowing for more accurate and comprehensive analyses

Estimated coefficients for are shown in the table for deviance, which is defined as the difference between the fitted model's log‐likelihood and the saturated model's log‐likelihood, is a number of the extent that the predictive algorithm fits to the facts. A model that has one parameter for every observation is known as a saturated model, and it fully fits the data.^21^ To assess the connection between platelet, count and other components of blood, one approach would be to fit a Poisson or negative binomial regression model with platelet count as the response variable and other blood components as predictors. Next, the fitted model's deviation can be computed and contrasted with a saturated model's deviance^22^ (Table 3).

**Table 3:** Model selection criteria.

Count model	AIC	BIC	Deviance
Poisson regression model	344.6474	364.6478	1344.2023
Negative binomial regression model	76.5494	76.5979	3.1484
Quasi‐Poisson regression model	202.3558	202.35867	202.3589

The negative binomial regression model appears to be the best‐fitting model, as it has the smallest AIC and BIC values and the lowest deviance. The Poisson regression model has much higher AIC and BIC values, indicating poorer fit, while the quasi‐Poisson regression model has extremely high AIC and BIC values, suggesting that it is not a good fit for the data.

The negative binomial model is often used when there is overdispersion in the data, which means that the variance is greater than the mean, and this appears to be the case here since the Poisson model did not fit the data well. Overdispersion is taken into consideration by the negative binomial model by introducing an extra parameter, which allows the variance to be greater than the mean.

Table 2 shows the outputs of different count regression models, including Poisson regression, negative binomial regression, and quasi‐Poisson regression, for platelet count and different WBC types (neutrophil, monocyte, lymphocyte, ESR, and eosinophils). For all three models, the intercept is substantial, suggesting that the platelet count deviates significantly from zero. While the Negative Binomial regression model only demonstrates significant effects for platelet count and neutrophils, the Poisson regression and quasi‐Poisson regression models for the WBC types reveal significant impacts for all kinds with *p*‐values less than 0.05 or 0.1. Although the WBC types' coefficients vary significantly throughout models, their effects are essentially the same. In conclusion, all three models can be used to predict platelet count and WBC types, but the quasi‐Poisson regression model may be preferred in cases of overdispersion.

### 3.4 Residual analysis of the negative binomial regression model

**Figure 2:**
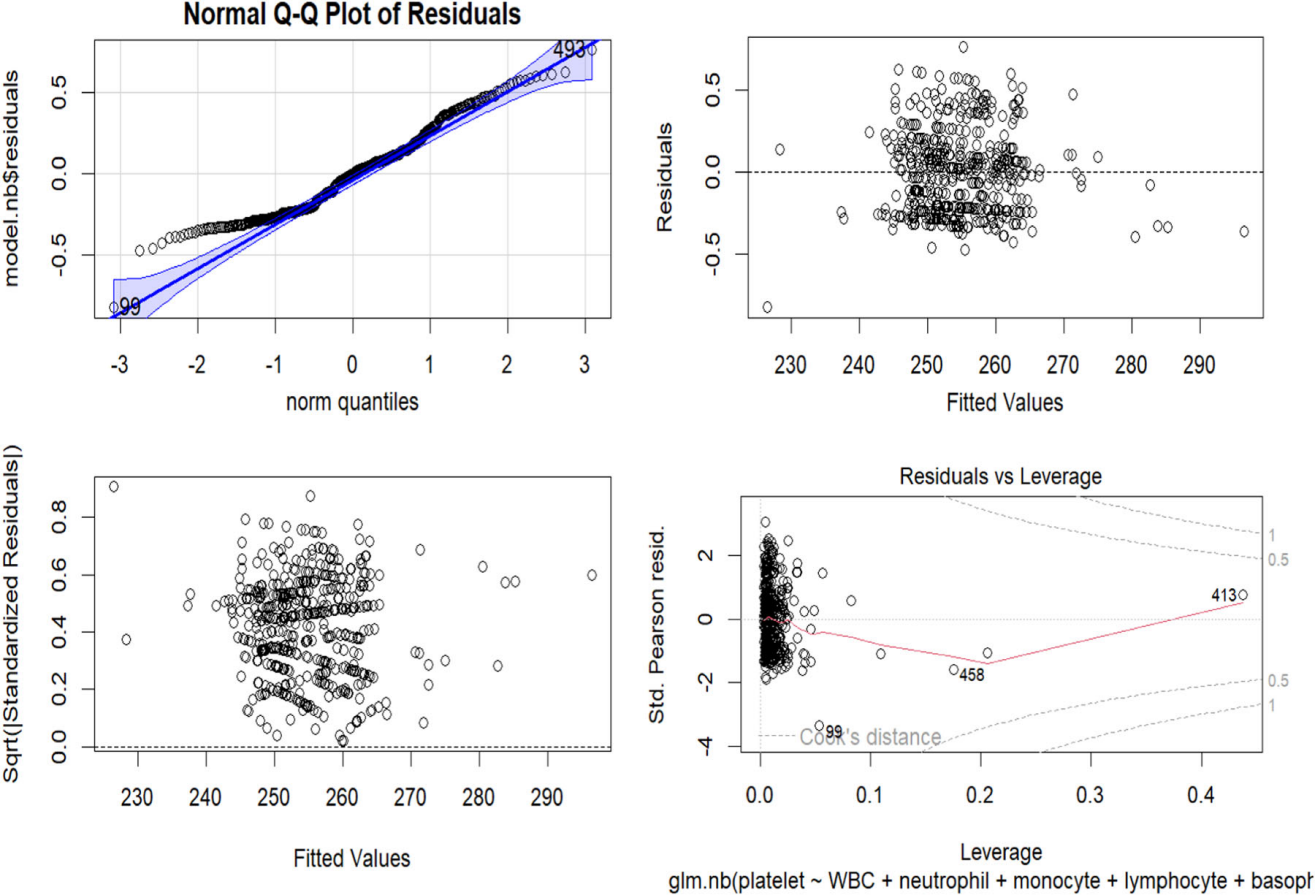
Residual analysis of the negative binomial regression model.

Figure 2 The residual plot evaluates the adequacy of the negative binomial regression model. The residuals versus fitted plot shows residuals against fitted values. Ideally, residuals should be randomly scattered around the zero line with no clear patterns. However, this plot reveals a concave‐up pattern, suggesting the model may not perfectly fit the data and might miss some nonlinearity between the predictors and the outcome variable. The quantile‐quantile (Q‐Q) plot compares theoretical quantiles of the standardized residuals with observed quantiles. Ideally, the points should form a straight line, indicating normally distributed residuals. However, the points deviate from the straight line, especially at the tails, indicating the residuals may not be perfectly normally distributed and suggesting the model may underestimate or overestimate the outcome variable's variability. The scale‐location plot compares the corresponding square fundamental of the normalized residuals to the estimated values. Ideally, every point should be arranged at random around a line that is horizontal, showing ensuring the variance left over remains stable at all predictor thresholds. In this plot, the points form a slight funnel shape, suggesting increasing variance of residuals with higher fitted values, implying the model may underestimate the outcome variable's variability at higher predictor levels. The residuals versus leverage plot shows leverage values against standardized residuals. Ideally, points should be randomly scattered with no clear patterns. This plot highlights one observation (Number 47) with a high leverage value and a large standardized residual, indicating it may be an influential point affecting the model's estimated coefficients. This observation should be further examined to determine if it is an outlier or genuinely affects the outcome variable.

## 4 DISCUSSION

Findings depict that, negative Binomial model was found to be the best fit and showed significant associations between platelet count and some WBC types. This has clinical significance for detecting and tracking medical diseases that influence platelet or WBC data. This research highlights the importance of adequate statistical frameworks to examine count data and suggests using advanced modeling techniques for further research. The study investigated the connection between platelet count and different types of WBCs using three different count regression models. The negative binomial model was found to be the most appropriate based on its statistical measures. Another study revealed that blood product ratios in pediatric trauma patients, revealing that higher ratios of fresh frozen plasma to packed red blood cells at 4 and 24 h after injury were connected with a lower risk of death at 24 h. Multivariable Poisson regression analysis was used to determine these findings. Additional prospective studies are needed to identify the optimal blood product ratios for reducing mortality in this population.^23^

Platelet count is significantly associated with all types of white treatment strategies and lessening the likelihood of patient complications blood cells in the Poisson and quasi‐Poisson models, while the negative binomial model showed significant association with some types of WBCs. The choice of regression model can influence the conclusions drawn about the connection between platelet count and WBC types. The best fit compared to Poisson and negative‐binomial regression analyzing CBC tests including platelet counts, various statistical methods have been used.^11^

## 5 FUTURE RESEARCH

In future efforts to gain a deeper comprehending of the connection between platelet count and other types of WBCs, researchers can employ sophisticated modeling approaches such as random forests or deep‐learning neural networks. They can also evaluate the impact of different variables including age, gender, and past medical treatment on the connection between platelet count and blood components, as well as investigate the clinical importance of these findings in individuals with certain medical disorders. This study has the potential to improve the diagnosis, treatment, and monitoring of these illnesses by providing fresh insights into the underlying mechanisms and potential hazards linked to differences in platelet along with WBC counts.

## 6 STRENGTH AND SIGNIFICANCE AS WELL AS LIMITATION

This work contributes to the expanding corpus of research on blood analysis and shows how useful count regression models are for figuring out how platelet count and other blood components relate to one another. The study emphasizes the value of combining qualitative and quantitative data collection approaches to acquire a thorough understanding of complicated medical diseases in terms of non‐communicable disease, as well as the importance of ongoing healthcare research. The study did have certain limitations, though, as biases, missing data, and unaccounted‐for factors that affect platelet count must be taken into account. To increase the generalizability of the findings, future studies should aim to replicate similar findings across larger and more diverse patient populations.

## 7 RECOMMENDATION

The results of the study have significant ramifications for medical professionals and researchers who work with blood analysis. The study sheds light on the relationship between platelet count and other blood components, perhaps helping clinicians better understand the underlying causes of numerous blood‐related disorders and devise efficient treatment techniques.

## 8 CONCLUSION

The connection between different types of WBCs and platelet count shows potential for forecasting platelet count, which can help with diagnosis and monitoring health issues. The connection between different types of WBCs and platelet count. The Negative Binomial regression model is preferred because it can account for higher variance in data than the mean. These discoveries are expected to aid healthcare practitioners by expediting diagnosis. Advanced modeling tools can help us understand the intricate interplay between platelet count and other WBCs, which could have practical significance for optimizing treatment options in disorders involving platelet or WBC counts.

## AUTHOR CONTRIBUTIONS

**Umme Honey**: Conceptualization; methodology; investigation; data curation; writing—review and editing; writing—original draft; formal analysis. SM **Arif Bin Saleh**: Conceptualization; investigation; supervision; data curation; writing—review and editing. **Md Sifat Ar Salan**: Conceptualization; methodology; software; formal analysis; supervision; writing—review and editing. **Mohammad Alamgir Kabir**: Conceptualization; validation; investigation; supervision; project administration; writing—review and editing. **Akher Ali**: Methodology; writing—original draft; data curation; formal analysis.

## CONFLICT OF INTEREST STATEMENT

The authors declare no conflict of interest.

## ETHICS STATMENT

The study obtained logistical clearance from the esteemed Department of Statistics at Jahangirnagar University, Savar, Dhaka‐1342, under reference code JU/STAT/EC/2022/170. Participation in the research was contingent upon the willing consent of individuals, signified by their acknowledgment through a formal permission form, ensuring ethical and informed analysis. Before taking part in the poll, all participants provided informed consent. They also consent to the publication of the survey's analytical results without their identifiable information.

## TRANSPARENCY STATEMENT

The lead author Md. Sifat Ar Salan affirms that this manuscript is an honest, accurate, and transparent account of the study being reported; that no important aspects of the study have been omitted; and that any discrepancies from the study as planned (and, if relevant, registered) have been explained.

## Data Availability

The data that support the findings of this study are available on request from the corresponding author. The data are not publicly available due to privacy or ethical restrictions. The data utilized in this study cannot be shared due to restrictions imposed by institutional policy and privacy regulations. However, reasonable requests for data access will be considered upon contacting the corresponding author.
